# Off-pump coronary artery bypass in poland syndrome with dextrocardia: case report

**DOI:** 10.1186/1749-8090-6-75

**Published:** 2011-05-18

**Authors:** Vivek Srivastava, Ranjit More, Augustine Tang

**Affiliations:** 1 Department of Cardiothoracic Surgery, Lancashire Cardiac Centre, Victoria Hospital, Blackpool, FY3 8NR, UK; 2 Department of Cardiology, Lancashire Cardiac Centre, Victoria Hospital, Blackpool, FY3 8NR, UK

**Keywords:** Coronary artery bypass graft surgery, CABG, Off-pump surgery, OPCAB, Pectus excavatum, Poland Syndrome

## Abstract

Poland Syndrome is a congenital disorder characterised by hypoplasia of the pectoral muscles along with upper extremity deformities. We encountered a patient with Poland syndrome associated with dextrocardia and also failed pectus excavatum repairs who presented to us with symptomatic ischaemic heart disease requiring intervention. He underwent successful off-pump coronary artery bypass surgery (OPCABG). As far as we are aware, this is the first case report of OPCABG in a case of Poland syndrome with dextrocardia. We describe here the management of this complex patient and wish to emphasise that the off-pump option is feasible in dextrocardia with some technical modifications.

## Off-Pump Coronary Artery Bypass in Poland syndrome with dextrocardia: Case Report

Poland syndrome (PS) is a rare congenital disorder with an incidence of 1 in 7,000 to 1 in 100,000 [1] characterized by hypoplasia of the pectoral muscles with associated upper extremity deformities. The anomalies in PS are attributed to hypoplasia of the subclavian artery or its branches as the result of an in-utero vascular accident [2,3]. Dextrocardia is an associated anomaly and has been reported in 5.6% cases of a series of 144 and in 9.6% of these, the defect was left-sided [2]. We encountered a case of left sided Poland syndrome associated with dextrocardia who presented to us with coronary artery disease and successfully underwent off-pump coronary artery bypass grafting (OPCABG).

## Case Summary

**Figure 1:**
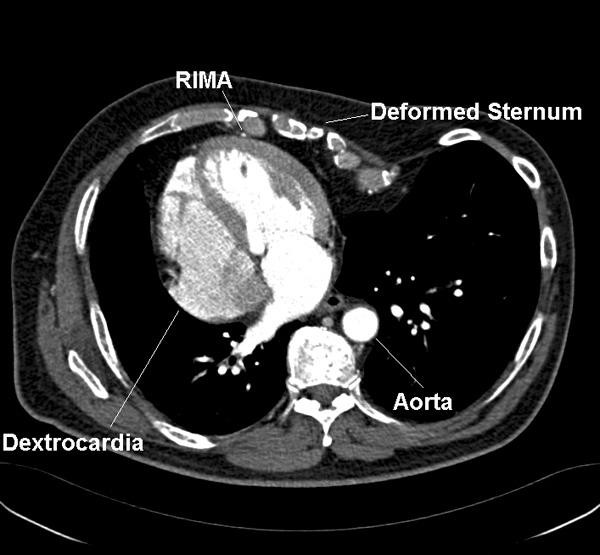
**CT scan showing dextrocardia with situs solitus**. (RIMA- Right Internal Mammary Artery).

A 66-year-old male presented with a two-month history of CCS class II angina. He had a history of two failed repairs of pectus excavatum - at twelve years age through midline and through the left chest at age of nineteen years. He had complete absence of the left pectoral muscle and a deformity of the left hand raising the diagnosis of Poland syndrome. Investigations revealed isolated dextrocardia with a concordant heart (situs solitus) (Figure 1). Coronary angiogram revealed proximal left anterior descending artery (LAD), proximal circumflex, mid right coronary artery (RCA) and ostial posterior descending artery (PDA) stenoses with preserved left ventricular function. Preoperative spirometry showed only mild airflow obstruction.

## Procedure

**Figure 2:**
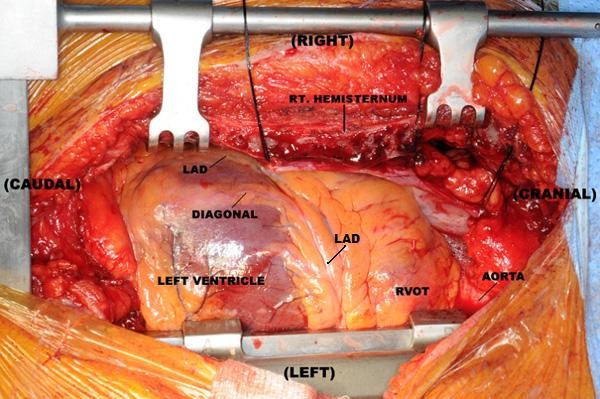
**Operative photograph demonstrating major cardiac mass under the right hemisternum.** The Left Anterior Descending Artery (LAD) is seen coursing from left to right. (RVOT - Right Ventricular Outflow Tract).

Following median sternotomy and pericardiotomy, the pericardial space was freed from adhesions. The apex of the left ventricle was seen under the xiphisternum and the left pulmonary artery was located near the ventral midline. Thus the lateral wall of the left ventricle was in full view in its native position (Figure 2). The right internal mammary artery (RIMA) was harvested as a pedicled conduit without any damage (which was technically very difficult because of the severe chest wall deformity) and had an excellent flow. The long saphenous vein (SVG) was harvested from the left leg. Following adequate heparinisation, two deep pericardial sutures were placed below the right phrenic nerve. The heart was then verticalised into the apex to ceiling position. The mid-LAD was intramyocardial - a target site in the distal LAD was therefore immobilised using the Octopus 4 (Medtronic Inc., Minneapolis, MN, USA) stabiliser. This was associated with a drop in the systolic blood pressure and the cardiac index despite small boluses of vasoconstrictor and inotrope. It was therefore decided to use an intra-aortic balloon pump (IABP) with subsequent hemodynamic stability. With the operating surgeon on the left side of the patient, the pedicled RIMA was anastomosed to a distal LAD arteriotomy with use of an intracoronary shunt. The surgeon then moved from the left side to the right side of the patient and inserted two left-sided deep pericardial sutures to maximise exposure of the obtuse marginal (OM) target. This was necessary because of the severe asymmetrical sternal deformity from pectus excavatum. The diagonal and then OM were grafted from the left side using SVG as usual. Again the surgeon moved to the right side to construct the PDA anastomosis using SVG. The proximal anastomoses were then constructed. Following full protamine reversal, the wound was closed as usual with steel wires for the sternum and the patient transferred to ICU. The IABP was removed on the 1^st ^postoperative day and the patient was discharged home on the 5^th ^day after an uneventful recovery.

## Discussion

Ailiwadi et al [4] have previously reported coronary artery bypass in a patient with Poland syndrome and demonstrated normal flow through the left internal mammary artery (LIMA) before using it as a conduit. In a recent article, Saad et al [5] reviewed coronary artery bypass in dextrocardia. They found 10 off-pump cases while 14 cases used cardiopulmonary bypass. In 16 of the 24 cases, the RIMA was grafted to the LAD. Surgery was performed from the right side in 5 cases and from the left in 10. The surgeon needed to switch sides in 3 cases. To our knowledge, ours is the first report of OPCABG in a case of Poland syndrome with dextrocardia and only the second case report of coronary artery bypass in Poland syndrome. Any concerns about an insufficient LIMA were addressed by use of RIMA. Surgery was further facilitated by a change in the surgeon's position. We wish to emphasise that OPCABG is a feasible option in patients with dextrocardia and adequate revascularisation can be achieved with planning and certain technical modifications.

## Declaration

Written informed consent was obtained from the patient for publication of this case report and accompanying images. A copy of the written consent is available for review by the Editor-in-Chief of this journal.

## Competing interests

The authors declare that they have no competing interests.

## Authors' contributions

VS was involved in the preparation of draft and finalisation of the manuscript. RM advised regarding preparation of manuscript. AT was the chief surgeon and responsible for finalisation of the manuscript.

All authors read and approved the final manuscript.
